# A Universal Oligonucleotide Microarray with a Minimal Number of Probes for the Detection and Identification of Viroids at the Genus Level

**DOI:** 10.1371/journal.pone.0064474

**Published:** 2013-05-29

**Authors:** Yongjiang Zhang, Jun Yin, Dongmei Jiang, Yanyan Xin, Fang Ding, Ziniu Deng, Guoping Wang, Xianfeng Ma, Fang Li, Guifen Li, Mingfu Li, Shifang Li, Shuifang Zhu

**Affiliations:** 1 Chinese Academy of Inspection and Quarantine, Beijing, China; 2 School of Medicine and Medical Science, Conway Institute, University College Dublin, Belfield, Dublin, Ireland; 3 State Key Laboratory of Biology of Plant Diseases and Insect Pests, Institute of Plant Protection, Chinese Academy of Agricultural Sciences, Beijing, China; 4 College of Plant Science and Technology, Huazhong Agricultural University, Wuhan, China; 5 Horticulture and Landscape College, Hunan Agricultural University, Changsha, China; Nanjing Agricultural University, China

## Abstract

A major challenge in the agricultural industry is the development of techniques that can screen plant samples for viroid infection. Microarrays are promising in this regard, as their high throughput nature can potentially allow for the detection of a range of viroids in a single test. In this paper we present a microarray that can detect a wide spectrum of all 8 reported viroid genera including 37 known plant viroid species. The array was constructed using an automated probe design protocol which generated a minimal number of probes to detect viroids at the genus level. The designed microarray showed a high specificity and sensitivity when tested with a set of standard virus samples. Finally, the microarray was applied to screen infected field samples, with *Hop stunt viroid* infection identified as the major disease causing pathogen for an infected citrus sample.

## Introduction

Viroids are plant pathogens with circular and single stranded RNA genomes. Viroid RNAs are short and not protein-coding. Crops affected by viroids may develop severe diseases, which may result in significant economic losses to the agricultural industry. Viroids can be spread worldwide via mechanical contact, seed transmission, or biological vectors. International trade also increases the chance of spreading the viroids to new territories. Thus a fast and accurate diagnostic tool is needed to control the disease and reduce the risk of the introduction of new viroids. Many diagnostic techniques have been developed for plant viroid detection. These include bioassays [1]–[4], return-polyacrylamide gel electrophoresis (Return-PAGE) [5], reverse transcription-polymerase chain reaction (RT-PCR) [6]–[15], RT-PCR using genus degenerate primers and multiplex primers [16]–[30], RT-PCR dot-blot hybridization (RT-PCR-DBH) [31], real time RT-PCR [32]–[37], molecular hybridization [38]–[40], reverse transcription loop-mediated isothermal amplification (RT-LAMP) [41], [42] and microarrays [43]–[47].

The aforementioned techniques can detect one or several viroid species or genera, but fail to detect a wide range of viroid pathogens. These techniques usually need *a priori* knowledge of the viroids and this makes them unsuitable to detect new emerging viroids. These emerging viroids may include new viroid species, as well as established viroid species being transferred to new host species or new territories.

Advances in next generation sequencing (NGS) technologies have provided a powerful alternative for pathogen detection. Using small RNA (sRNA) library construction and deep sequencing, NGS is able to identify both known and novel viroid species, and de novo assemble viroid genomes. Li et al. applied sRNA deep sequencing to analyze infected tomato samples and detected *Potato spindle tuber viroid* (PSTVd), *Pepino mosaic virus (*PepMV) and an unknown potyvirus species [48]. Several computational algorithms were developed to detect the viroids quantatively using deep sequencing [49] or in a homology independent manner [50]. However, NGS is very computation intensive and much more expensive than the commonly used methods, e.g. PCR or ELISA.

Thus there is a great need for a high throughput, cost effective and relative specific way of detecting plant viroid pathogens. Microarrays with genus level probes are suitable tools for this purpose. Using genus level probes, microarrays are able to detect multiple viroid genera in a single chip and are capable of detecting new viroids at the genus level. Several microarrays have been reported that detect a wide range of viruses [51]–[54]. However, no microarray with a similar capability of detecting plant viroids has been reported before.

In this paper, we report a new microarray platform which detects plant viroids at the genus level. A minimal number of 40 mer genus level probes were selected for 36 species from all the 8 reported plant viroid genera and 1 unclassified viroid species. Several standard plant viroid samples were collected and validated using this microarray. The application of the microarray was tested on three infected plant samples from the field. *Hop stunt viroid* was detected as the major causative pathogen for an infected citrus sample.

## Materials and Methods

### Viroid Standard Samples and Nucleotide Sequences

Standard viroid samples were collected by the Institute of Plant Protection, Chinese Academy of Agricultural Sciences (Beijing, China), and purchased from the American Type Culture Collection (Manassas, VA, USA) and several other sources (Tables 1 and 2). These viroid samples were verified by sequencing. The sequence of *Coleus blumei viroid* collected by the Institute of Plant Protection, Chinese Academy of Agricultural Sciences was submitted to the GenBank database with accession number of KC581915.

**Table 1:** Plant viroid sequences obtained from the NCBI Taxonomy Browser and used to design the 40-mer oligonucleotide probes for the microarray.

Viroid family	Viroid genus/species	No. of species^a^	No. of genome sequences	No. of nucleotide sequences	No. of probes
*Avsunviroidae*	*Avsunviroid*	1	1	101	5
	*Elaviroid*	1	1	10	4
	*Pelamoviroid*	2	2	756	9
*Pospiviroidae*	*Apscaviroid*	11^b^	10	655	35
	*Cocaviroid*	4	4	55	9
	*Coleviroid*	6	6	26	6
	*Hostuviroid*	1	1	418	8
	*Pospiviroid*	10	9	544	19
Unclassified	*Apple fruit crinkle viroid*	1	1	94	8
	Total	37	35	2659	103

a according to NCBI taxonomy browser.

b although several nucleotide sequences were downloaded for *Australian grapevine viroid*, no probe was designed for this species.

**Table 2:** Viroid samples used to test the performance of the microarray.

Family	Genus	Species	Provider	Samples
*Avsunviroidae*	*Avsunviroid*	*Avocado sunblotch viroid* (ASBVd)	ATCC	Plant tissue (PV-663)
	*Pelamoviroid*	*Chrysanthemum chlorotic mottle viroid* (CChMVd)	ATCC	Plant tissue (PV-120)
	*Pelamoviroid*	*Peach latent mosaic viroid* (PLMVd)	Beijing CIQ	Plant tissue
*Pospiviroidae*	*Apscaviroid*	*Apple scar skin viroid* (ASSVd)	CAAS-IPP	Plant tissue
	*Apscaviroid*	*Citrus dwarfing viroid* (CDVd)	HUNAU	Plant tissue
	*Cocadviroid*	*Hop latent viroid* (HLVd)	CAAS-IPP	Plant tissue
	*Coleviroid*	*Coleus blumei viroid 1* (CbVd-1)	CAAS-IPP	Plant tissue
	*Hostuviroid*	*Hop stunt viroid* (HSVd)	CAAS-IPP	Plant tissue
	*Pospiviroid*	*Chrysanthemum stunt viroid* (CSVd)	CAAS-IPP	Plant tissue
	*Pospiviroid*	*Citrus exocortis viroid* (CEVd)	HZAU	Plant tissue
	*Pospiviroid*	*Columnea latent viroid* (CLVd)	ATCC	Plasmid (45122)
	*Pospiviroid*	*Potato spindle tuber viroid* (PSTVd)	CAAS-IPP	Plant tissue
	*Pospiviroid*	*Tomato apical stunt viroid* (TASVd)	ATCC	Plasmid (45053)
	*Pospiviroid*	*Tomato planta macho viroid* (TPMVd)	ATCC	Plasmid (45052)

CAAS-IPP: Chinese Academy of Agricultural Sciences,The Institute of Plant Protection (Beijing, China).

HZAU: Huazhong Agricultural University (Huzhong, China).

HUNAU: Hunan Agricultural University (Hunan, China).

ATCC:American Type Culture Collection (Manassas, VA, USA).

Beijing CIQ: Beijing Entry-Exit Inspection and Quarantine Bureau (Beijing, China).

A total of 35 full-length genomic sequences and 2,659 partial genomic sequences were downloaded from the NCBI Taxonomy Browser (http://www.ncbi.nlm.nih.gov/Taxonomy/) (Table 1). These sequences come from eight viroid genera. Nucleotide sequences shorter than 250 bp were removed. If there were more than 100 sequences in a viroid genera, nucleotide sequences with >98% identical nucleotide across >98% of the sequence were clustered and only the longest sequence was retained as the representative sequence for the following analysis. This left 421 filtered sequences to be used in the probe design. An additional 943 plant viral genome sequences and 105 895 plant mRNA sequences were used to identify off-target hybridizations.

### Microarray Probe Design

A probe design protocol was applied to generate a minimal number of genus level probes as described in [54]. Non-redundant viroid nucleotide sequences within the same genus were aligned using BLASTN [55]. Conserved regions were identified from the alignment and searched for 40 nt probes. The candidate probes were required to (i) have a GC content between 40 and 60%, (ii) be less than four continuous mononucleotides, and (iii) have less than six nucleotides in the inner hairpin. Probes showing potential off-target hybridization to the other viroid genera, plant viruses and plant mRNA were removed. Probes that passed the above filters were aligned to all the sequences in the viroid genus using BLASTN. A probe to viroid sequence match was defined as at least 80% of the probe matching the viroid sequence. Final microarray probes were selected by maximizing the coverage of viroid genera using a minimal number of probes. Selection of probe combination was repeated three times, thus three probe sets were generated to target most viroid sequences by three probes.

### Microarray Construction

Microarrays were synthesized using the selected 40 mer probes on 75-mm×25-mm glass slides using the SmartArrayer system following the manufacturer’s instruction (CapitalBio, Beijing, China) (Table S1). All probes were spotted in triplicate on the microarray. Several control probes were used to monitor the performance of the microarray. (i) The 5′ amino-linker oligonucleotide (5′-TTTTTTTTTTTTTTTCTCATGCCCATGCCGATGC-3′) was a random sequence which did not potentially hybridize with any viroid sequences. The targeted sequence of this probe was mixed with the samples before hybridization. This probe was used as positive control (PC) monitoring the probe hybridization efficiency. (ii) The internal control (HEX) was spotted on the microarray to monitor the probes linking to the chips. It was the 5′ Hex-linker oligonucleotide (5′-GTCACATGCGATGGATCGAGCTCCTTTATCATCGTTCCCACCTTAATGCA-3′). (iii) Probes designed to match the highly conserved regions in plant 18 s rRNAs were used as positive controls to detect host plant rRNAs. (iv) The spotting buffer was used as the negative control (NC). In a standard hybridization result, the PC and HEX probes should be positive with high signal intensities. NC probes should be negative with signal intensities similar to the background level. In this study, the viroid sequences were amplified using species specific primers, thus the quantity of plant rRNA were very low in the cDNA library. The plant rRNA probes should be negative in the hybridization results.

### Viroid cDNA Synthesis and PCR Amplification

Total RNA was extracted from plant samples using TRIzol reagent (Invitrogen, Carlsbad, CA) following the manufacturer’s protocols. RNA was purified using the NucleotideSpin® RNA clean-up (MN, Duren, Germany).

Reverse-transcription (RT) reactions were performed using viroid species degenerate primers (Table 3). In brief, 1 µl of total RNA was mixed with 2 µl of 20 µM primers, 1 µl of 10 mM dNTPs and 10 µl of RNase free water, denatured at 70°C for 5 min and quickly chilled on ice for 5 min. Then, 6 µl of reverse transcription mix containing 4 µl of 5X reverse transcription buffer, 1 µl of 200 U/µl M-MLV reverse transcriptase and 1 µl of 40 U/µl RNase inhibitor (Promega Corporation, WI, USA) were added to a final volume of 20 µl. The tubes were incubated at 42°C for 1 h for viroid cDNA synthesis.

**Table 3:** PCR primers used to verify the standard viroid samples.

		Viroid	Primers	Sequence (5′–3′)	Amplified DNA (bp)	Reference
*Avsunviroidae*	*Avsunviroid*	ASBVd	ASBVd f	AGTTCACTCGTCTTCAATCTC	247	This work
			ASBVd r	CTGAAGAGACGAAGTGATCAA		
	*Pelamoviroid*	CChMVd	CChMVd f	GGCACCTGATGTCGGTGT	399	This work
			CChMVd r	GACCTCTTGGGGGTTTCAAAC		
	*Pelamoviroid*	PLMVd	PLMVd f	CCAGGTAACGCCGTAGAAACTG	337	[59]
			PLMVd r	ATCACACCCTCCTCGGAACCAA		
*Pospiviroidae*	*Apscaviroid*	ASSVd	ASSVd f	CCGGATCCGGTAAACACCGTGCGGTCCC	330	[60]
			ASSVd r	CCGGATCCGGGAAACACCTATTGTGTTT		
	*Apscaviroid*	CDVd	CDVd f	GGCAGCTAAGTTGGTGACGC	296	[25]
			CDVd r	TTCGTCGACGACGACAGGTA		
	*Cocadviroid*	HLVd	HLVd f	ACCTACTCGAGCGAGGCGGAG	256	This work
			HLVd r	GCACGAACTGGCGCTCGAT		
	*Coleviroid*	CbVd-1	CbVd-1f	TTGGATCCAGCGCTGCAACGGAATCCA	250	[5]
			CbVd-1r	TTGGATCCGCCAGGGAACCCAGGTAAG		
	*Hostuviroid*	HSVd	HSVd f	GGGGAAATCTCGAGTTGCCG	285	This work
			HSVd r	GATCCGCGGCAGAGGCTT		
	*Pospiviroid*	CSVd	CSVd f	CGGGACTTACTTGTGGTTCC	354	This work
			CSVd r	AGGGAACAAAACTAAGGTTCCAC		
	*Pospiviroid*	CEVd	CEVd f	GGAAACCTGGAGGAAGTCGAG	370	[61]
			CEVd r	CCGGGGATCCCTGAAGGACTT		
	*Pospiviroid*	CLVd	CLVd f	GGATCCCCGGGGAAACCT	370	This work
			CLVd r	CTGAAGCGCTCCTTTGGC		
	*Pospiviroid*	PSTVd	PSTVd f	CGGAACTAAACTCGTGGTTCCTGTGG	359	[62]
			PSTVd r	AGGAACCAACTGCGGTTCCAAGG		
	*Pospiviroid*	TASVd	TASVd f	AAGCTTCCACTTCCACGCTC	360	This work
			TASVd r	CAGTTGTTTCCACCGGGTAGT		
	*Pospiviroid*	TPMVd	TPMVd f	GGATCCCCGGGGAAACCT	360	This work
			TPMVd r	CTGAAGCGCTCCTTTGGC		

The PCR reaction mix contained 2 µl cDNA or plasmid, 2 µl 10X PCR buffer, 0.5 µl of 20 µM forward primer, 0.5 µl of 20 µM reverse primer, 0.5 µl of 10 mM dNTPs, 0.5 µl of 5 U/µl *Taq* polymerase and 14 µl of RNase free water (20 µl in total). PCR was performed at 94°C for 5 min, followed by 35 cycles at 94°C for 0.5 min, 55∼58°C according to different primer pairs for 0.5 min and 72°C for 1 min. A final elongation step was performed at 72°C for 7 min.

### Microarray Fluorescence Labeling and Hybridization

Fluorescence labeling reactions were performed with a volume of 25 µl. 5 µl of PCR product was mixed with 2 µl of 20 µM nonamer random primers and 12 µl of RNase free water, denatured at 95°C for 3 min and then quickly chilled on ice for 5 min. Then, it was mixed with 2.5 µl of 10X Klenow fragment buffer, 2 µl of 10 mM dNTPs, 0.5 µl of 25 nM cy3-dCTP and 1 µl of 5 U/µl Klenow fragment. The tubes were incubated at 37°C for 1.5 h and 70°C for 5 min. The fluorescence labeling product hybridization, microarray wash, image acquisition and signal analyses were performed as described previously [54].

The sensitivity of the microarray was evaluated using the leaf of a *Humulus lupulus* sample infected with *Hop stunt viroid* (HSVd). The concentration of the total RNA was determined using a UV spectrophotometer as 200 ng/µl. The RNA was serially diluted 10^0^, 10^1^, 10^2^ and 10^3^ times, and used in the RT-PCR and microarray hybridization. The hybridization results of the three dilutions were compared to determine the microarray detection sensitivity.

The microarray data were submitted to the Gene Expression Omnibus (GEO) database with the platform accession number of GPL16684 and series accession number of GSE44334.

### Virus Identification

Viroid genus and species were identified using the novel microarray using a revised protocol of that previously described [54]. Positive probes were selected as those with a feature signal intensity more than three times that of the background intensity, and a feature intensity minus background intensity greater than 1500. The signal strength of a genus is the sum of signal intensities of all the positive probes in this genus. The signal strength was converted to relative signal strength by dividing by the maximum signal strength of all the genera. The relative signal strength is represented as a percentage, for example, 1 or 100%, and used to rank genera. The viroid genus with the highest relative signal strength is predicted as the major viroid genus infecting the plant. The relative signal strength of a species is calculated using the same principle as the genus calculation; dividing the sum of the signal intensities of all the positive probes in a species by the maximum sum of the signal intensities of positive probes of all the species. The viroid species with the highest relative signal strength is predicted as the major species infecting the plant.

### Screening Field Samples

To assess the performance of the microarray when screening field samples, several plants showing disease symptoms were collected. A tomato sample and a chrysanthemum sample were collected from Beijing, China. A citrus sample was obtained from Hunan Province, China. Microarray hybridization and data analysis protocols were the same as described above. Collection of the field samples followed the regulations of the Chinese Academy of Inspection and Quarantine. No endangered or protected species was involved.

## Results

### Construction of the Microarray

Microarray probes were designed matching the most conserved regions of viroid sequences. A minimal number of probes per genus were selected as described in the Materials and Methods. Approximately 2,000 nucleotide sequences were downloaded from the NCBI sequence database, which included all the 36 reported viroid species in 8 genera and 1 unclassified species. A total of 103 genus level probes were designed, which are able to target 36 out of all the 37 viroid species (Tables 1 and S1). Only *Australian grapevine viroid* did not have any probes designed. On average, each genus is targeted by 11.4 probes and each species is targeted by 2.8 probes. 29 out of the 37 viroid species were targeted by more than three probes. This enabled detection of viroids to the species level. In addition, control probes monitoring probe ligation and hybridization efficiency were used as described in the Materials and Methods.

### Evaluation of the Microarray Performance

**Figure 1:**
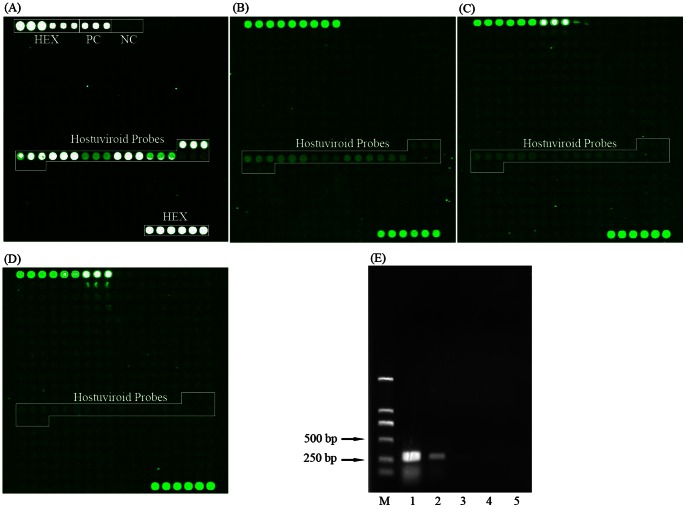
Sensitivity test of HSVd. Microarray hybridization pseudo-color images were generated for RNA dilutions of (A) 10^0^, (B) 10^1^, (C) 10^2^ and (D) 10^3^ fold. Control probes and probes targeting *Hostuviroid* are highlighted with rectangles. (E) RT-PCR was performed for different RNA dilutions and a negative control. HEX was used as an internal control to monitor ligation efficiency. PC, positive control. NC, negative control.

The microarray detection sensitivity was evaluated using the total RNA isolated from *Humulus lupulus* leaves infected with *Hop stunt viroid* (HSVd). Total RNA was extracted and quantified by UV spectrophotometer as 200 ng/µl. 10^0^ to 10^3^ folds dilutions of the total RNA were screened using the microarray. The top genus and species predicted for RNA dilutions of 10^0^ and 10^1^ were *Hostuviroid* and HSVd, respectively (Figure 1). No *Hostuviroid* probes gave positive signal at dilutions of 10^2^ and 10^3^. Similar results were observed by RT-PCR amplicons on agarose gel. Only RNA dilutions of 10^0^, 10^1^ and 10^2^ were visible on the gel. This indicates that at least 20 ng/µl total RNA is required for accurate and specific microarray identification.

14 standard viroid samples from 7 genera were collected and tested using the microarray (Tables 2 and 4). 13 out of 14 samples were correctly identified at the genus level, while 12 out of 14 samples were correctly identified at the species level. Only *Hop latent viroid* showed contradictory result in the genus level, and was predicted as the second most possible pathogen with 70% signal strength. Instead, one *Apscaviroid* probe showed unspecific signal.

**Table 4:** Microarray prediction for the standard viroid samples.

Standard viroid samples			Genus prediction score		Contradictory genus prediction			Species prediction score		Contradictory species prediction		
Family	Genus	Species	Rank	Relative Signal	Genus	Rank	Relative Signal	Rank	Relative Signal	Species	Rank	Relative Signal
*Avsunviroidae*	*Avsunviroid*	*Avocado sunblotch viroid*	1	1				1	1			
	*Pelamoviroid*	*Chrysanthemum chlorotic mottle viroid*	1	1				1	1			
	*Pelamoviroid*	*Peach latent mosaic viroid*	1	1				1	1			
*Pospiviroidae*	*Apscaviroid*	*Apple scar skin viroid*	1	1				1	1			
	*Apscaviroid*	*Citrus dwarfing viroid*	1	1				1	1			
	*Cocadviroid*	*Hop latent viroid*	2	0.7	Apscaviroid	1	1	2	0.7	*Pear blister canker viroid*	1	1
	*Coleviroid*	*Coleus blumei viroid 1*	1	1				1	1			
	*Hostuviroid*	*Hop stunt viroid*	1	1				1	1			
	*Pospiviroid*	*Chrysanthemum stunt viroid*	1	1				1	1			
	*Pospiviroid*	*Citrus exocortis viroid*	1	1				1	1			
	*Pospiviroid*	*Columnea latent viroid*	1	1				1	1			
	*Pospiviroid*	*Potato spindle tuber viroid*	1	1				1	1			
	*Pospiviroid*	*Tomato apical stunt viroid*	1	1				1	1			
	*Pospiviroid*	*Tomato planta macho viroid*	1	1				2	0.67	*Citrus exocortis viroid*	1	1

**Figure 2:**
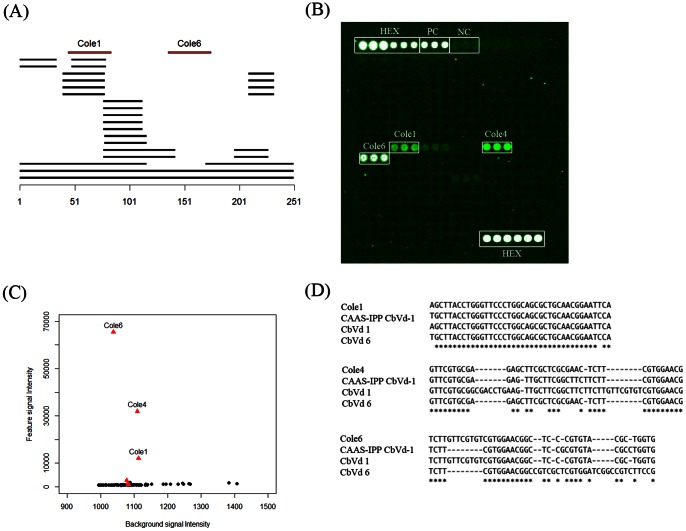
Probe design and microarray detection of *Coleviroid*. (A) Two oligonucleotide probes Cole1 and Cole6 were designed matching conserved regions of *Coleus blumei viroid 1*. Black bars are high-scoring segment pairs (HSP) from the BLASTN alignments of the other *Coleviroids* to *Coleus blumei viroid 1* (X95291). Red bars are the alignments of Cole1 and Cole6 to *Coleus blumei viroid 1*. (B) Pseudo-color image of *Coleus blumei viroid 1* hybridization on the microarray. All probes with feature signal intensity minus background signal intensity more than 1500 are marked on the images. HEX, positive control (PC) and negative control (NC) are marked on the image. (C) Feature and background signal intensities of all the viroid probes on the microarray. Probes targeting *Coleviroid* genera are depicted as red triangles. Other probes are depicted as black circles. (D) Sequence alignment of *Coleviroid* probes Cole1, Cole4 and Cole6 with the *Coleus blumei viroid 1* (KC581915) sequenced by CAAS-IPP, and *Coleus blumei viroid 1* (CbVd 1, X95291) and *Coleus blumei viroid 6* (CbVd 6, NC_012805) genomic sequences from NCBI.

*Coleus blumei viroid* is selected as an example to illustrate the probe design and microarray detection workflow. Nucleotide sequences from *Coleviroid* genus were aligned using BLASTN to identify conserved sequences. As shown in Figure 2A, probe Cole1 was designed to match the most conserved region of *Coleus blumei viroid* 1, with more than 9 high-scoring segment pairs (HSP) aligned from different *Coleviroid* species. Cole6 is another probe targeting *Coleus blumei viroid* 1. The targeting region of probe Cole6 is less conserved than Cole1, however, this enables more specific species identification using Cole6 (Figure 2A, D). In the microarray hybridization results, Cole1, Cole4 and Cole6 were identified as positive probes (Figure 2B). These *Coleviroid* probes exhibit highly specific feature when compared to background signals (Figure 2C). No cross hybridization to probes in other genera were detected. Interestingly, probe Cole4 is designed to detect *Coleus blumei viroid* 5 and 6 variants, and shares low sequence similarity to *Coleus blumei viroid* 1. This suggests the *Coleus blumei viroid* sample used in the microarray hybridization may be a new variant more similar to *Coleus blumei viroid 1* and shares certain sequence features to variant 5 and 6. A further sequencing of the *Coleus blumei viroid* sample confirmed the high sequence similarity, 87%, to the published *Coleus blumei viroid 1* genome sequence (Figure 2D). However, it also revealed similar sequence features of the *Coleus blumei viroid* sample to *Coleus blumei viroid 6*, as targeted by Cole4 on the microarray.

### Screening Field Samples

**Figure 3:**
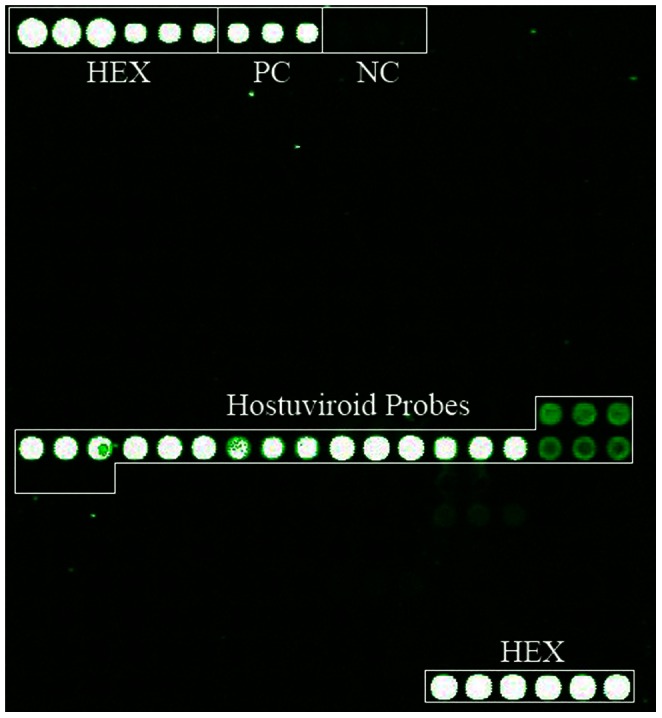
Microarray hybridization pseudo-color image of the infected citrus sample. Eight probes were designed for *Hostuviroid* and spotted on the microarray in triplicate. These probes are highlighted on the image. Seven out of the eight *Hostuviroid* probes were positive. *Hop stunt viroid* was predicted as the major pathogen of the infected citrus sample. No other viroid probe was positive on the microarray. HEX, positive control (PC) and negative control (NC) are marked on the image.

In order to test the application of the microarray, several plant samples were collected from the field showing disease symptoms of unknown pathogenic cause. These field samples were screened using the viroid microarray (Figure 3 and Table 5). A citrus sample showing bark scaling on rootstock and dwarfing was found to have a *Hop stunt viroid* infection (also known as *Citrus viroid II*). In the microarray hybridization results, 7 out of 8 probes from *Hostuviroid* showed strong specific hybridization signals. *Hop stunt viroid* was predicted as the major causative pathogen. Further sequencing of the viroid showed an identical sequence to the published *Hop stunt viroid* genome.

**Table 5:** Microarray screening of field samples with disease symptoms.

Field samples	Disease symptom	Genus prediction score					Species prediction score				
		Genus	Rank	Relative Signal	No. of Positive Probes	No. of AllProbes	Species	Rank	Relative Signal	No. of Positive Probes	No. of All Probes
Citrus	bark scaling	*Hostuviroid*	1	1	7	8	*Hop stunt viroid*	1	1	7	8
Tomato	yellow leaf curl symptom	–	–	–	–	–	–	–	–	–	–
Chrysanthemum	yellow and stunt symptom	–	–	–	–	–	–	–	–	–	–

## Discussion

Detection of viroid infection is a major challenge in plant disease diagnosis and control. The existing methods, such as molecular hybridization and PCR, are labor intensive and less efficient when screening for a wide range of known viroids [56], [57]. To overcome the shortage of these methods, we have developed a new plant viroid detection microarray.

Currently, 31 plant viroid species in 8 genera and 1 unclassified viroid species are reported in the International Committee on Taxonomy of Viruses (ICTV). 5 additional viroid species were deposited in NCBI as newly discovered variants. Previously, only a few microarrays were reported to detect a subset of plant viroids. A recently published microarray was able to detect six viroid species from the *Pospiviroid* genus [45]. A commercial microarray was constructed that is able to detect 7 viroid genera [47]. However, the microarray developed in this study is able to detect 35 viroid species out of the 36 reported species from all 8 reported genera plus an unclassified viroid species.

A major focus in microarray design is the selection of probes. The short viroid genome sequences limits the choice of probe selection. Though long oligonucleotide probes (> = 70 bp) may provide more consistent hybridization signals [58], only 3 to 4 non-overlapping 70 bp sequences can be selected from a viroid genome. Thus a shorter probe with 40–50 bp is a better choice. An appropriate number of probes per viroid should be designed to achieve consistent results. A large number of probes may provide higher statistical power, but increase the cost and redundancy of the microarray. In contrast, a small number of probes may lack the consistency and sensitivity required to detect emerging viroids with novel sequence variations. A minimum of three 40 mer probes per viroid is recommended to ensure the consistent detection of nucleotide sequences [58]. Thus in our study, the probe design protocol was optimized to select a minimal number of 40 mer probes and retain three probes per viroid to reach a balance of probe efficiency and detection specificity.

In the sensitivity test of the microarray, an RNA dilution of 1∶10 was detected by microarray. While the microarray showed a negative result for the dilution of 1∶100, a weak signal of 1∶100 dilution was detected using RT-PCR. Thus refinement of the microarray amplification procedure is needed to increase the sensitivity of detection in the future.

14 standard viroid samples from 7 genera were collected to validate the performance of the microarray. 13 out of 14 samples were successfully identified at the genus level, with a detection accuracy of 92.9%. The probes were designed matching the most conserved region in a genus to maximize the coverage of the viroid species. Because of the short genome sequence and large sequence variation of viroids, probes only detecting viroids at the species level were included in order to improve the probe coverage. This enabled the detection of viroids to the species level by using a species specific combination of probes. Using the standard viroid samples, 12 out of 14 samples were accurately identified at the species level, with an accuracy of 85.7%. In the sample infected with *Tomato planta macho viroid*, *Citrus exocortis viroid* was predicted as the disease causing pathogen. *Tomato planta macho viroid* and *Citrus exocortis viroid* are from the same genus, *Pospiviroid*. The false prediction in the species level was due to shared probes between these two species. In the future, including more species specific probes may increase the accuracy of the microarray at the species level.

The viroid screening of field samples using our microarray was particularly interesting. No positive probes were identified in the microarray screening of infected tomato and chrysanthemum samples. These samples might be infected by plant viruses. A combination of plant virus microarray [54] and viroid microarray may improve the success of pathogen screening. In the infected citrus sample, *Hop stunt viroid* was detected as the major infectious pathogen, which was consistent with a traditional RT-PCR test [25]. This highlights the usefulness of microarrays in detecting viroid pathogens in field plants.

In conclusion, we have developed a 40 mer oligonucleotide microarray for the detection and identification of all 8 reported plant viroid genera and 36 out of 37 reported species. This microarray detected the largest number of plant viroids reported so far. A minimal number of probes were used to reduce the cost and probe redundancy of the microarray. Validation of the microarray using standard viroid samples showed consistent and reliable results. This diagnostic tool would be useful in screening and identification of viroid infections in plant disease discovery and control.

## Supporting Information

Table S1 Annotation of probes on the viroid detection microarray.
(XLSX)
