# Correction: A Universal Oligonucleotide Microarray with a Minimal Number of Probes for the Detection and Identification of Viroids at the Genus Level

**DOI:** 10.1371/annotation/4a3a55a5-a298-4d9f-ad42-3c0a9a2e50e1

**Published:** 2013-10-24

**Authors:** Yongjiang Zhang, Jun Yin, Dongmei Jiang, Yanyan Xin, Fang Ding, Ziniu Deng, Guoping Wang, Xianfeng Ma, Fang Li, Guifen Li, Mingfu Li, Shifang Li, Shuifang Zhu

A funding organization and multiple grants were incorrectly listed in the Funding Statement.

In addition, multiple funding organizations and grants were incorrectly omitted from the Funding Statement. The Funding Statement should read: "This research was supported by AQSIQ Program (2010IK278), MOST Programs (2012BAK11B00) and the Irish Research Council for Science Engineering and Technology (IRCSET) Graduate Education Programme (GREP). The funders had no role in study design, data collection and analysis, decision to publish, or preparation of the manuscript." 

